# Book Notices

**Published:** 1868-09

**Authors:** 


### Book Notices.

A Therapeutical and Practical Treatise on Midwifery; Including the Diseases of Pregnancy and Parturition. By P. CAZEAUX, Member of the Imperial Academy of Medicine; Adjunct Professor in the Academy of Medicine of Paris; etc., etc., etc. Adopted by the Superior Council of Public Instruction. Revised and Annotated by S. TARNIER, Adjunct Professor in the Faculty of Medicine of Paris, Hospital Surgeon, etc. Fifth American from the Seventh French Edition. By W. R. BULLOCK, M.D. With One Hundred and Seventy-Five Illustrations. Philadelphia: LINDSAY & BLAKISTON. 1868. For sale by COBB, PRITCHARD, & CO., Chicago.

Cazeaux's Midwifery has been so long before the profession, and its merits have been so well appreciated, both in this country and Europe, that comments are unnecessary. The present edition is made more valuable by the notes and additions by Tarnier, and is published in excellent style, with a copious and convenient index.

---

A Manual on the Extraction of Teeth. Founded on the Anatomy of the Parts Involved in the Operation; the kinds and proper construction of the Instruments to be used; the Accidents liable to occur from the Operation, and the proper Remedies to retrieve such Accidents. By ABRAHAM ROBERTSON, D.D.S., M.D., Author of Prize Essay on Extracting Teeth; Fellow of the N.H. Medical Society, etc., etc., etc. Second Edition. Philadelphia: LINDSAY & BLAKISTON. 1868.

This is an elegantly published duodecimo volume of 200 pages. It will be found very useful to dentists and all physicians who engage in the extraction of teeth.

---

Dental Materia Medica. Compiled by JAMES W. WHITE. Philadelphia: SAM'L S. WHITE. 1868.

This is a small duodecimo volume of 108 pages, neatly printed and bound; and, from a hasty glance at its contents, we should regard it as a work of real value and convenience to every practical dentist, and not without interest to the physician.

---

The Anatomy and Histology of the Human Eye. By A. METZ, M.D., Professor of Ophthalmology in Charity Hospital Medical College, Cleveland, Ohio. Philadelphia: Office of Medical and Surgical Reporter. 1868.

This is an octavo volume of 184 pages. The mechanical execution of the work is all that could be required. The paper, type, binding, and illustrations are decidedly good. From a hasty examination, we should say that the author had also executed his task well. His descriptions of the anatomy and histology of all the structures concerned in the organs of vision are clear, concise, and yet full. For the student, it constitutes an excellent text-book; and for the ophthalmologist and general practitioner, a very convenient work for reference.

---

Half-Yearly Compendium of Medical Science. Part II. July, 1868. Philadelphia: S. W. BUTLER, M.D., 115 S. 7th St.

This is the second number of the Half-Yearly Compendium of Medical Science. It contains 208 large-sized pages, with a copious index. It contains 420 articles, selected from American and foreign medical periodicals. It is published for $3 per annum, or $2 for a single copy. The numbers are issued in January and July. All those practitioners who are not able to subscribe for more than one or two medical periodicals will find the *Compendium* a very valuable work for reference.

---

Goff's Combined Day-Book, Ledger, and Daily Register of Patients, for the Use of Physicians.

This is a volume of 328 pages, substantially bound, and so ruled as to fill perfectly all the purposes of a day-book and ledger, with space for memoranda, and a copious index. It presents every man's account for a year, both in detail and aggregate, debit and credit on the same page, ready for examination at all times, without the trouble of handling different books or posting from one to another. The book is admirably arranged to save time and promote convenience in keeping accounts, and we commend it to the attention of the profession.

